# Air pollution, mortality, and hospital admissions in Scotland: A 16 years register-based study

**DOI:** 10.23889/ijpds.v8i2.2195

**Published:** 2023-09-14

**Authors:** Mary Abed Al Ahad, Urška Demšar, Frank Sullivan, Hill Kulu

**Affiliations:** 1 University of St Andrews, St Andrews, United Kingdom

## Objectives

Air pollution is associated with poor health and higher mortality. However, studies that link high spatial resolution air pollution data for several pollutants to individual-level data over prolonged period (>10 years) and assess multiple health outcomes are limited. In this study, we investigated the association between 16-years exposure to air pollution and all-cause and cause-specific (cardiovascular, respiratory, cancer, infectious, and mental/behavioural disorders) mortality and hospital admissions in Scotland.

## Method

Individual-level data from the “Scottish Longitudinal Study” for 202,237 individuals (2002-2017) were linked to yearly concentrations of NO2, SO2, PM10, and PM2.5 pollutants at 1-Km2 spatial resolution using the individual’s residential postcode. The association between air pollution and mortality and hospital admissions was examined using Cox Proportional-Hazards and multilevel mixed-effects negative binomial models, respectively.

## Results

Increasing concentrations of PM2.5, PM10, and NO2 pollutants were associated with higher rates of all-cause, cardiovascular, respiratory, cancer, and infectious mortality and hospital admissions. Mortality from respiratory diseases increased by 6.2% (95%CI=2.8%-9.6%), 2.5% (95%CI=0.5%-4.5%), and 1.2% (95%CI=0.5%-1.9%) per 1 µg/m3 increase in PM2.5, PM10 and NO2 pollutants, respectively. Exposure to SO2 was mainly linked to mental/behavioural disorders mortality (HR=1.05; 95%CI=1.02-1.07) and respiratory hospital admissions (IRR=1.02; 95%CI=1.01-1.03).

## Conclusion

This study revealed a positive association between air pollution and mortality and hospital admissions in Scotland. Interventions on air pollution through stricter environmental regulations could help ease the mortality and hospital admission burden, for both physical and mental illness.

